# Proficiency test on the determination of polyethylene and polybutylene terephthalate cyclic oligomers in a food simulant

**DOI:** 10.1016/j.fpsl.2019.100441

**Published:** 2020-03

**Authors:** Emmanouil D. Tsochatzis, Joao Alberto Lopes, Pieter Dehouck, Piotr Robouch, Eddo Hoekstra

**Affiliations:** European Commission, Joint Research Centre, Via Enrico Fermi 2749, Ispra VA, 20127, Italy

**Keywords:** Food contact materials (FCM), Polyethylene terephthalate (PET) cyclic oligomers, Polybutylene terephthalate (PBT) oligomers, Proficiency test (PT), Food simulant D1

## Abstract

The outcome of a proficiency test (PT) organised by the European Union Reference Laboratory for Food Contact Materials (EURL-FCM) is presented. The PT was set up to assess the analytical performance of National Reference Laboratories (NRLs) and Official Control Laboratories (OCLs) in the determination of mass fractions of polyethylene terephthalate (PET) and polybutylene terephthalate (PBT) cyclic dimers and trimers in the official food simulant D1 containing ethanol and water (50:50 v/v).

The EURL-FCM had developed and validated an analytical method based on HPLC-UV to monitor the homogeneity and stability of the target oligomers in the PT test items and to determine the respective assigned values, as prescribed in ISO 17043, 2010ISO 17043, 2010. The standard operating procedure of the method was provided to the participants and could be used instead of their own routine methods. Laboratory results were rated using z, z' and ζ scores in accordance with ISO 13528, 2015. The standard deviation for proficiency assessment, *σ_pt_*, was set to 20 % of the respective assigned value, for all the four studied oligomers, based on the perception of experts.

A total of 36 participants from 26 countries have registered to the exercise. They received two test items. Solution 1 consisted of food simulant D1 fortified with a known mass fraction of the four oligomers, while Solution 2 was obtained by a migration experiment with PET bottles and food simulant D1 and further fortification of the resulting solution with the four oligomers.

The majority of the participating laboratories presented satisfactory results for the four PET and PBT oligomers. For the analysis of Solution 1, 79–88 % of the participants obtained |z (or z')-scores| below 2, while the satisfactory performances ranged from 71 to 85 % for Solution 2. This PT has been organised for the first time at EU level for the analysis of polyester oligomers and confirms that most of the NRLs are able to monitor properly these oligomers in the frame of Regulation (EU) No 10/2011.

## Highlights

• Migration of food contact material substances.
• Mass fractions of PET and PBT cyclic dimers/trimers in food simulant D1.
• First proficiency test on polyester oligomers.
• Validation of analytical method.
• Assessment of analytical capabilities of EU NRLs and OCLs.

## 1 Introduction

Polyesters (PES) belong to a group of plastics that are commonly used in many applications. This group represents ca. 18 % of the world polymer production (Wu, Hu, Du, Jiang, & Yu, 2014). For food contact materials (FCMs), polyethylene terephthalate (PET) is the preferred polyester due to its impact resistance, strength, flexibility and resistance to high temperatures (Hoppe, Fornari, de Voogt, & Franz, 2017; Wu et al., 2014). In addition polybutylene terephthalate (PBT) is currently used for the production of other FCMs, e.g. coffee capsules, oxygen-barrier films, kitchenware, microwaveable dishware, drinking mugs and other beverage containers (Brenz, Linke, & Simat, 2017; Brenz, Linke, & Simat, 2018).

Regulation (EU) No 10/2011 sets maximum specific migration limits (SMLs) for all additives and monomers allowed to be used in plastic FCM (EU, 2011). PET oligomers are considered as non-intentionally added substances (NIAS), and can potentially migrate from the plastic material to the food, similar to polyamide and polystyrene oligomers (Genualdi, Nyman, & Begley, 2014; Heimrich, Nickl, Bönsch, & Simat, 2015; Kappenstein et al., 2018). However no SMLs have been established for NIAS. A mixture of cyclic PBT oligomers (FCM No 885) from the dimer up to the pentamer is already authorised and included in the positive list of Regulation (EU) No 10/2011 (EFSA, 2009; EU, 2011). The use of this mixture has been approved as an additive in PET, PBT, polycarbonate, polystyrene and rigid poly(vinyl chloride) (EU, 2011) with mass fractions up to 1 % w/w for contact with aqueous, acidic and alcoholic foods and for long-term storage at room temperature.

Toxicological data for most of the existing plastic packaging oligomers are scarce and their risk for human health remains largely unclear. No legislative limits are in place for their individual migration into food, but there are a few migration limits for the sum of oligomers with a mass below 1000 Da. In order to contribute to a better understanding of the migration of PET and PBT oligomers and to a reliable measurement of these oligomers, the EURL-FCM organised the first European proficiency test (PT) under ISO 17043 accreditation (ISO 17043, 2010ISO 17043, 2010) for national and official control laboratories in 2018.

The EURL-FCM selected the 1^st^ series PET cyclic dimer and trimer and the PBT cyclic dimer and trimer due to their relevance in the FCM field. The selected substances are the main components of classes of oligomers with a mass below 1000 Da, of particular toxicological interest.

This paper summarises the outcome of the first PT focusing on assessing the measurement capabilities of control laboratories of the FCM network for the determination of the mass fractions of PET and PBT cyclic dimers and trimers in food simulant D1 solutions (EU, 2011, 2017).

## 2 Materials and methods

### 2.1 Chemicals

Ethanol (EtOH), acetonitrile (ACN) and 1,1,1,3,3,3-hexafluoro-2-propanol (HFIP) were of Chromasolv grade, supplied by Sigma Aldrich (Steinheim, Germany). Ultrapure water (MilliQ, Millipore, Darmstadt, Germany) was used in the preparation of solutions and simulants. While the PET cyclic dimer and trimer were supplied by TRC Chemicals (Toronto, Canada), the PBT cyclic dimer and trimer have been isolated from a raw polymeric mixture provided by industry and purified with an automated preparative High Performance Liquid Chromatography with system. The purity of the resulting PBT oligomers was analysed by Nuclear Magnetic Resonance (NMR) and High-resolution Mass Spectrometry (HR-MS). This work has been described elsewhere (Tsochatzis et al., 2018).

**Fig. 1:**
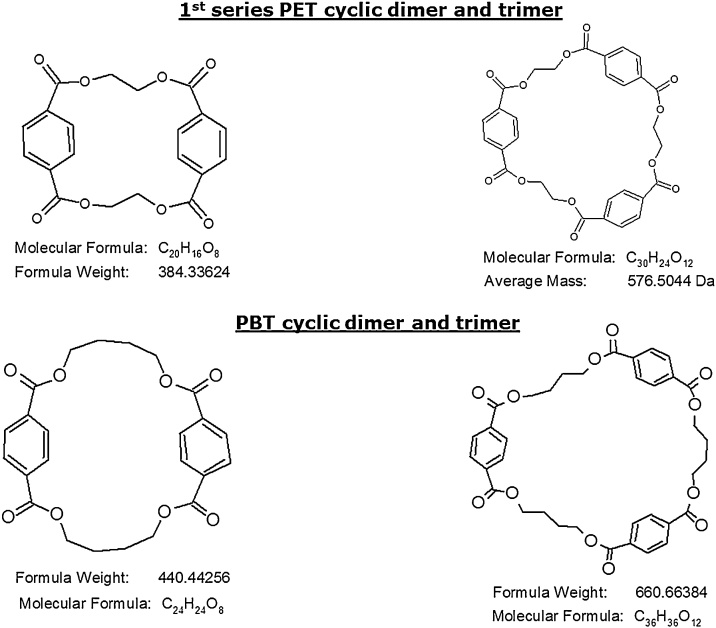
Chemical structures of studied PBT and 1^st^ series and PET cyclic oligomers.

In addition, the EURL-FCM investigated the solubility of the four oligomers in various solvents and found HFIP as most effective in solubilising equally all the studied substances. Therefore HFIP was used to prepare the required stock solutions. All the structures of the studied PET 1^st^ series and PBT cyclic dimers and trimers are presented in Fig. 1.

### 2.2 Preparation of the test solutions

Food simulant D1 (ethanol:water 50:50 v/v) was prepared according to Annex III of Commission Regulation (EU) No 10/2011 (EU, 2011). Half a litre of this solution was gravimetrically fortified with the four cyclic dimers and trimers of interest and mixed for 5 min to obtain Solution 1. For the preparation of Solution 2, a virgin PET bottle was used to perform first a migration experiment. The bottle was kindly supplied by ALPLA-Werke Lehner GmbH & Co. KG (Vlotho, Germany), shipped on a cardboard base covered with polyethylene, and stored at room temperature (max. 25 °C) after reception. The migration experiment was performed with 500 mL of food simulant D1 at a temperature of 70 ± 2 °C for 2 h. After the migration experiment the solution was gravimetrically fortified with the four cyclic dimers and trimers) and mixed for 5 min to obtain Solution 2. Portions (5 mL) of Solutions 1 and 2 were transferred separately into 25 mL screw capped Schott bottles and stored at -18 °C. In addition, a stock solution of the cyclic oligomers was prepared gravimetrically in HFIP/EtOH (9:1 v/v) at a concentration of 20 μg mL^-1^. Portions (1 mL) were manually transferred into 5 mL amber vials and stored at -18 °C. Each bottle and vial was identified with a unique number and the name of the PT exercise.

A dedicated feasibility study was performed (i) to evaluate the effective fortification of food simulant D1 and to optimise the preparation of the test materials for the PT; (ii) to assess the stability of the material at different temperatures for a certain period of time; and (iii) to investigate any potential interferences from the glass containers (vial or bottle).

### 2.3 Sample dispatch and instructions

Each participant received two bottles containing 5 mL of test Solution 1 and Solution 2, repectively, and a vial with 1 mL of the oligomer stock solution. The “Sample accompanying letter” specified the measurands of interest as "the mass fractions (mg kg^-1^) of PET cyclic dimer, PET cyclic trimer, PBT cyclic dimer and PBT cyclic trimer in food simulant D1". Laboratories were requested to perform two or three independent measurements on each test item and to report the mean, the associated expanded measurement uncertainty (mg kg^-1^) with its coverage factor (k) and the analytical technique used to perform the measurements. The measurement results had to be corrected for recovery. Participants were asked to follow their routine procedures for the analysis and to report results in the same way (e.g. number of significant figures) as they would report to their customers.

A temperature sensor/indicator was included in the parcel to check that the temperature of the cooled parcel remained below 4 °C during the whole shipment.

### 2.4 Analytical method validation

A dedicated method based on HPLC with optical detection (λ = 240 nm) was developed and validated to monitor the homogeneity and stability of the four selected PET and PBT cyclic oligomers in the two test items (Solutions 1 and 2) and to determine the corresponding assigned values with associated measurement uncertainties (ISO 17043, 2010ISO 17043, 2010). An Agilent Zorbax Eclipse XDB-C18 column (150 × 4.6 mm, 5 μm), thermostated at 40 °C (±1 °C) was selected. The mobile phase consisted of acetonitrile (solvent A) and water (solvent B). The elution was based on the following isocratic-linear gradient sequence: start at 40 % A (t=0 min); kept isocratic for 6 min; linear gradient to 75 % A (t=15 min); second linear gradient increase to 95 % A (t = 21 min); 3 min equilibration time to the initial conditions (40 % A). The injection volume was set to 50 μL.

The standard operating procedure (SOP) of the method mentioned above and the corresponding validation report were provided to participants as an alternative method which they could use instead of their own routine method.

### 2.5 Assigned values

The assigned values (*x_pt_*) for the four selected PET and PBT cyclic oligomers in the two solutions were determined by the EURL-FCM using the analytical method mentioned above. The intermediate precisions determined in the frame of the method validation study were set for each analyte as the standard uncertainty contributions due to characterization, *u_char_* (ISO 5725-3, 1994).

The associated standard uncertainties of the assigned values (*u(x_pt_*)*)* were calculated combining *u_char_* with the standard uncertainty contributions due to homogeneity (*u_hom_*) and stability (*u_st_*):

(1) $$u\left(x_{pt}\right)=\sqrt{u_{char}^{2}+u_{hom}^{2}+u_{st}^{2}}$$

The standard deviations for proficiency assessment (σ_pt_) for all the measurands were set to 20 % of the assigned values based on expert judgment (ISO 13528, 2015). Table 1 presents the assigned values (*x_pt_*) with associated expanded uncertainties (*U(x_pt_)*, calculated using a coverage factor *k* = 2 and corresponding to a 95 % confidence level) and the standard deviations for proficiency assessment (*σ_pt_*) for Solution 1 and Solution 2.

**Table 1:** Assigned value (*x_p_*_t_), associated expanded uncertainty U(*x_pt_*) and standard deviation for the PT assessment (*σ_pt_*) in tested solutions (all values expressed in mg kg^-1^) for the two solutions and the four oligomers.

Cyclic oligomer	PET dimer	PET trimer	PBT dimer	PBT trimer
**Solution 1**				
*x_pt_*	0.0550	0.0530	0.0538	0.0502
*U(x_pt_)*	0.0052	0.0052	0.0074	0.0122
*σ_pt_*	0.0110	0.0106	0.0108	0.0100
*u(x_pt_)/σ_pt_*	0.2	0.2	0.3	**0.6**
**Solution 2**				
*x_pt_*	0.0585	0.1645	0.0706	0.0509
*U(x_pt_)*	0.0056	0.0160	0.0098	0.0124
*σ_pt_ (% of x_pt_)*	0.0117	0.0329	0.0141	0.0102
*u(x_pt_)/σ_pt_*	0.2	0.2	0.3	**0.6**

### 2.6 Scores and evaluation criteria

The individual laboratory performance was expressed in terms of z and ζ scores (ISO 13528, 2015).

(2) $$z=\;\frac{x_{i}-x_{pt}}{\sigma_{pt}}$$

(3) $$\zeta =\;\frac{x_{i}-x_{pt}}{\sqrt{{u(x_{i})}^{2}+{u(x_{pt})}^{2}}}$$

where:

*x_i_* is the measurement result reported by a participant;

*u(x_i_)* is the standard measurement uncertainty reported by a participant;

*x_pt_* is the assigned value;

*u(x_pt_)* is the standard measurement uncertainty of the assigned value and

*σ_pt_* is the standard deviation for proficiency assessment.

When *u(x_pt_)* > 0.3*σ_pt,_* as for the PBT cyclic trimer in the two solutions (see Table 1), the uncertainty of the assigned value (u(*x_pt_*)) was taken into account by expanding the denominator of the z score and calculating the z' score:

(4) $$z'=\;\frac{x_{i}-x_{pt}}{\sqrt{{\sigma_{pt}}^{2}+{u(x_{pt})}^{2}}}$$

The interpretation of the z (or z') and ζ scores was done according to ISO 17043, 2010ISO 17043, 2010 (ISO 17043, 2010ISO 17043, 2010), where |score| ≤ 2; 2 < |score| < 3; or |score| ≥ 3 correspond to satisfactory; questionable or unsatisfactory performances.

The *z* scores compare the participant's deviation from *x_pt_* with the standard deviation for proficiency assessment (*σ_pt_*) as a common quality criterion, while *ζ* scores report the agreement of a laboratory result with the assigned value taking into account the respective uncertainty. The *ζ* score includes the expected value (assigned value), its measurement uncertainty and the uncertainty of the reported values. A non-satisfactory *ζ* score could be caused by an inappropriate estimation of the mass fraction of the analyte, or of its measurement uncertainty, or both. The standard measurement uncertainty of the laboratory *u(x_i_)* was obtained by dividing the reported expanded measurement uncertainty by the reported coverage factor *k*. When no uncertainty was reported, it was set to zero (*u(x_i_)* = 0). When *k* was not specified, the reported expanded measurement uncertainty was considered as the half-width of a rectangular distribution; *u(x_i_)* was then calculated by dividing this half-width by √3, as recommended by Eurachem (Ellison & Williams, 2012).

A disadvantage of the *ζ* score is that an overestimation of the reported measurement uncertainty is not reflected in the score. Therefore, an additional evaluation has been performed comparing the reported measurement uncertainty *u(x_i_)* by the participating laboratories to the uncertainty on the assigned value *u(x_pt_)* (set as a minimum) and to the standard uncertainty for proficiency assessment *σ_pt_* (set as a maximum): *u(x_i_)* would most likely fall between *u(x_pt_)* and *σ_pt_* (case "a"). It is unlikely that a laboratory carrying out the analysis on a routine basis would determine the measurand with a smaller measurement uncertainty than the expert laboratories chosen to establish the assigned value. If *u(x_i_)* is smaller than *u(x_pt_)* (case "b") the laboratory may have underestimated its measurement uncertainty. However, such an assumption has to be taken with care as each laboratory reported only its measurement uncertainty, whereas *u(x_pt_)* also includes contributions for homogeneity and stability of the test item. If those are large, measurement uncertainties smaller than *u(x_pt_)* are possible and plausible. If *u(x_i_)* is larger than *σ_pt_* (case "c") the laboratory may have overestimated its measurement uncertainty. An evaluation of this assumption can be made when looking at the difference between *x_i_* and *x_pt_*: if the difference is smaller than the expanded uncertainty *U(x_pt_)* then an overestimation is likely. If the difference is larger but *x_i_* agrees with *x_pt_* within their respective expanded measurement uncertainties, then the measurement uncertainty is properly assessed resulting in a satisfactory performance (expressed as a ζ score) though the corresponding performance expressed as a z score may be questionable or unsatisfactory.

## 3 Results and discussion

### 3.1 Method validation

**Fig. 2:**
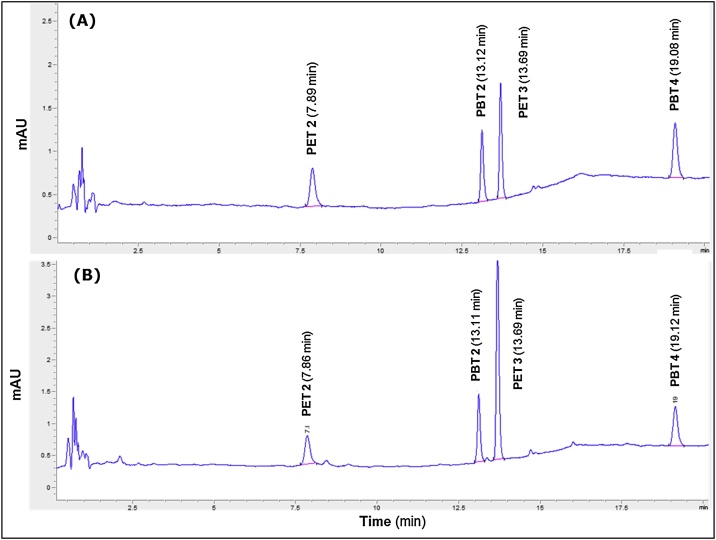
Examples of chromatograms of: (A) solution 1 of fortified food simulant D1 and (B) solution 2 fortified food simulant D1 after migration testing of a PET bottle.

The HPLC method described above was proven to be suitable for the accurate determination of the four target PET and PBT cyclic oligomers present at low mass fractions (μg kg^−1^) in food simulant D1. A typical chromatogram of a fortified food simulant D1 solution with 1.0 mg kg^-1^ is presented in Fig. 2 displaying well separated and clearly identified peaks: PET cyclic dimer at t_R_ =7.88 min; PBT cyclic dimer at t_R_ =13.14 min; PET cyclic trimer at t_R_ =13.71 min; and PBT cyclic trimer at t_R_ =19.14 min.

The selectivity of the method was demonstrated by resolution factors (R_s_) higher than 1.5 in all cases, indicating the good separation of all the target analytes. The working range was checked between 0.010 and 1.0 mg kg^-1^ and showed linear calibration curves characterized by regression coefficients above 99 % and residuals below 5 % with no significant trends identified for the four oligomers. Six replicate analyses were performed during three consecutive days, with a dedicated calibration curve per day. The single-factor analysis of variance (ANOVA) statistical test was used to evaluate the relative repeatability and intermediate precision standard deviations (s_r_ and s_ip_, respectively) which ranged from 1 to 6 % (higher values were obtained for the PBT trimer). For the assessment of the trueness, a blank solution of food simulant D1 (containing none of the target analytes) was spiked at three concentration levels. Recovery rates (R_rec_) ranging from 90 to 108 % were obtained (Table 2). Limits of detection (LOD) and quantification (LOQ) were evaluated from the chromatographic signal-to-noise ratio S/N (Table 2). Noise was calculated 30 s before the start of the peak and 30 after the end of the peak. Mean value and standard deviation of the S/N were obtained from 6 chromatograms of the lowest calibration level (0.010 mg kg^-1^). The LOD was calculated as 3 times the S/N, while the LOQ calculated as 3 times the LOD.

**Table 2:** Limits of detection and trueness results for short-term repeatability and intermediate precision of the validated HPLC method.

Short-term repeatability				
Added (mg kg^-1^)	Recovery			
	PET dimer	PBT dimer	PET trimer	PBT trimer
0.02	108.0 %	98.5 %	99.6 %	91.7 %
0.10	100.0 %	95.7 %	96.2 %	90.5 %
1.00	99.5 %	99.5 %	99.5 %	100.7 %

Intermediate precision				
Added (mg kg^-1^)	Recovery			
	PET dimer	PBT dimer	PET trimer	PBT trimer
0.02	108.0 %	100.0 %	96.5 %	94.5 %
0.10	102.0 %	96.0 %	95.0 %	90.5 %
1.00	100.7 %	100.3 %	100.3 %	99.7 %
**LOD** (mg kg^-1^)	0.005	0.003	0.002	0.003

The performance characteristics presented in Table 3 comply with the acceptance criteria recommended in the literature (Bratinova, Raffael, & Simoneau, 2009; Magnusson & Örnemark, 2014; Thompson, Ellison, & Wood, 2002).

**Table 3:** Precision characteristics of the validated HPLC method for short-term repeatability and intermediate precision.

	Mass fraction level (mg kg^-1^)	PET dimer	PBT dimer	PET trimer	PBT trimer
**s_r_**	0.02	3.0 %	6.0 %	3.7 %	6.5 %
	0.10	4.1 %	3.4 %	2.0 %	6.9 %
	1.00	2.3 %	2.3 %	2.0 %	12.1 %
**s_ip_**	0.02	3.4 %	6.9 %	4.9 %	9.0 %
	0.10	4.7 %	3.6 %	2.2 %	9.2 %
	1.00	2.6 %	3.1 %	2.3 %	12.2 %

### 3.2 Characterisation of the test items

The validated HPLC method was used to monitor the homogeneity and stability of the two solutions according to ISO 13528, 2015 (ISO 13528, 2015). The assessment of homogeneity was performed after the test items were vialled and before the distribution to participants. A random selection of 10 test items from Solution 1 and Solution 2 were analysed in duplicate and evaluated by a one-way ANOVA. Both solutions proved to be adequately homogeneous for all the measurands. The uncertainty contribution due to homogeneity (*u_hom_*) was calculated using SoftCRM (SoftCRM).

The initial feasibility studies revealed that all the oligomers were stable up to 20 °C, with exception of the PET cyclic dimer, which seemed to be more sensitive to hydrolysis at temperatures above 4 °C. This reaction happened at an even higher rate when temperatures were close to 20 °C. This may be attributed to the fact that PET, as well as PBT cyclic oligomers, are esters which are exposed to hydrolysis-promoting media (aqueous solutions). One way to avoid/decrease the possibility of hydrolysis after migration testing is to direct analyse such samples after testing. In any case it must be highlighted that in the official control of FCMs it is not a common practice to perform migration testing (i.e. at 70 °C for 2 h) with a food simulant (i.e. 50 % ethanol in water) and retain the sample for 2 months prior to the analysis. In such a case the hydrolysis of esters will eventually occur after a certain period of time, not only with the studied oligomers. It must be also assumed that hydrolysis of esters is not an automatic and immediate process, and for this prolonged storage of aqueous simulants after migration testing must be avoided.

But there are other potential promoters of hydrolysis. In this work was found that, after bottling, certain glass vials lead to much faster hydrolysis reaction of PET dimer. Different types and qualities of glassware were then evaluated to ensure the stability of the testing solutions during storing. After selecting a proper glass container, a preventive measure was also implemented to reduce hydrolysis: decreasing the storage temperature to ensure the stability of the solution throughout the period of the PT (3 months).

Hence, the test items were stored at -18 °C. The stability of all the oligomers was investigated at -18 °C and 4 °C, for a time period of 3 months, corresponding to the duration of the PT. The oligomers of interest proved to be sufficient stable at temperatures of -18 °C and 4 °C. The uncertainty contribution due to stability (*u_stab_*) was set to zero. It has to be noted that the stock solutions prepared in the HFIP/EtOH mixture did not show any hydrolysis even at 20 °C or higher.

**Fig. 3:**
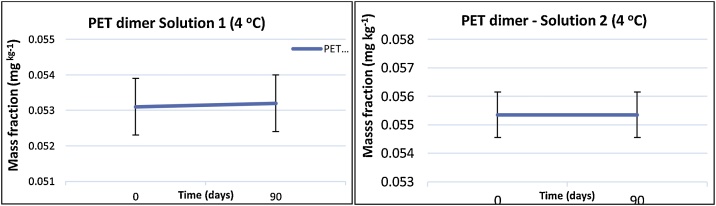
Results of the stability study for PET dimer in the two studied solutions (1 and 2) at 4 °C throughout the duration of the PT.

The stability study for the whole period of the PT, for each analyte at the two temperatures (+4oC, -18 oC), showed that there were no statistical differences in the mass fractions for all studied oligomers in compliance with ISO 13528 (ISO 13528, 2015), Thus, it could be concluded that both solutions were stable. An example for PBT dimer at +4 °C is shown in Fig. 3, where all the remaining analytes showed the same stable behaviour.

### 3.3 Assessment of the laboratory performances

A total of 36 laboratories from 26 countries registered to the PT FCM-18-01 (27 National Reference Laboratories (NRLs) and 9 Official Control Laboratories (OCLs)). All but one NRL and one OCL reported results for the four target oligomers in both test solutions, and answered the associated questionnaire. One laboratory reported a truncated value ("less than") for the PBT cyclic dimer in both solutions.

**Fig. 4:**
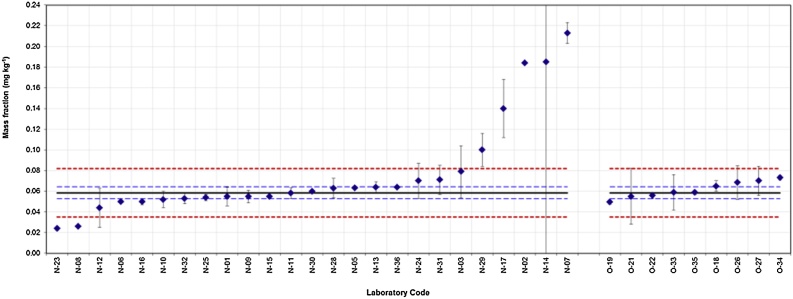
Results and associated expanded uncertainties reported by NRLs and OCLs (denoted N-xx or O-xx, respectively) for the determination of the mass fraction of PET cyclic dimer in food simulant D1. Reference value (*x_p_*_t_): solid black line; Reference interval (*x_pt_* ± U_(_*_xpt_*_)_): dashed blue lines; Target interval (*x_pt_* ± 2 *σ_pt_*):dotted red lines. (For interpretation of the references to colour in this figure legend, the reader is referred to the web version of this article.)

Fig. 4 presents a typical profile of collected results together with the respective expanded uncertainties. Most of the laboratory results fall between the red dotted lines indicating satisfactory z scores. Similarly, laboratories with a reported range (*x_i_* ± 2 *u*(*x_i_*)) overlapping the assigned range (*x_pt_* ± 2 *u*(*x_pt_*)) will have satisfactory ζ scores. Fig. 3 clearly shows that laboratory N-14 may have overestimated its measurement uncertainty.

**Fig. 5:**
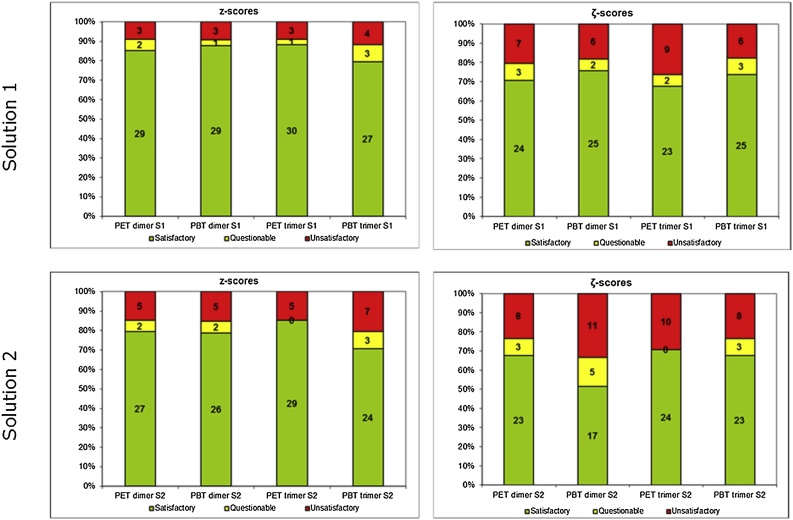
Laboratory performance according to *z* and *ζ* scores for Solution 1 and Solution 2 (satisfactory, questionable and unsatisfactory performances in green, yellow and red, respectively). (For interpretation of the references to colour in this figure legend, the reader is referred to the web version of this article.)

The overall performance of the participants regarding the z (z' for PBT cyclic trimer) and ζ scores is summarised in Fig. 5. It shows better laboratory performances for Solution 1 (spiked) than for Solution 2 (spiked after migration), with satisfactory performance ranging from 79 % to 88 % and from 71 % to 85 % for Solutions 1 and 2, respectively. This may be attributed to the presence of interfering migrated substances from the PET bottle in Solution 2. The percentage of satisfactory ζ scores was lower than the one of z or z' scores for all analytes. The biggest difference was found for the PBT cyclic dimer, with 79 % and 52 % satisfactory z and ζ scores, respectively.

The truncated values ("*less than* 0.04 mg kg^-1^") reported by one laboratory for the PBT cyclic dimer in both solutions correspond to the LOQ of the method applied. While these values could not be scored *per se* they were compared to the corresponding *x_pt_ – U(x_pt_)*. Since the truncated values were lower than "0.050 – 0.01" (mg kg^-1^) the reported statements were considered incorrect - the laboratory should have been able to quantify the analyte.

### 3.4 Evaluation of reported measurement uncertainties

Instead of assessing only the laboratory performance according to z, z' and ζ scores, the EURL-FCM offered also the possibility to benchmark the measurement uncertainty (MU) estimates. The following was observed: the majority of the laboratories (47 % or 16 out of 34) reported "underestimated" MU (case "b") calculated as the standard deviation of replicate measurements; 12 laboratories (35 %) provided "realistic" MUs (case "a"); two laboratories used the maximum acceptable MU of 25 %; and three laboratories did not report any MU; while one laboratory erroneously reported MU in % (instead of mg kg^-1^, e.g. 0.061 ± 10). None of the laboratories participating in this exercise had a validated method for the analysis of oligomers, and so they relied on non-validated in-house methods for which no precision data was available. This fact, combined with the lack of a proper estimation of the MU, could explain the observed large percentage of case "b" estimations.

**Fig. 6:**
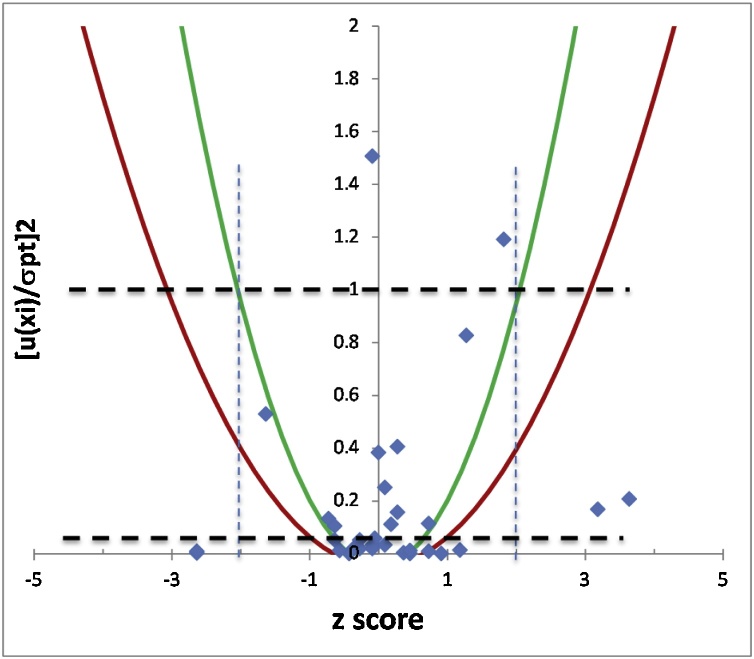
Naji plot for PT results on the PET cyclic dimer in Solution 1.

Fig. 6 presents the Naji plot (Frazzoli, Robouch, & Caroli, 2010) for the example of the PET cyclic dimer in food simulant D1 where the various conclusions are evidenced:

- The majority of the laboratories performed satisfactorily according to (i) the z score (29 out of 34 between the two vertical dotted lines delimiting |z|< 2) and (ii) to the ζ score (24/34 inside the green parabola delimiting |ζ|< 2). However, some of the satisfactory results (according to the z score) are flagged with questionable or unsatisfactory ζ scores (delimited by the green (|ζ| = 2) or red (|ζ| = 3) parabolas), mainly due to the underestimated measurement uncertainties reported.
- Few points are above the upper dashed horizontal line (*u*(*x_i_*) > *σ_pt_*) indicating likely "overestimated" MUs.
- Many points are below the lower dashed horizontal line (*u*(*x_i_*) < *u*(*x_pt_*)) highlighting "underestimated" MUs.

### 3.5 Information received from the participants

The questionnaire answered by all the participants provided additional information about the analytical methodologies used. Even though all participants except one stated to be accredited according to ISO 17025 (IEC/ISO 17025, 2017IEC/ISO 17025, 2017), most of them (30/34) had no experience with the analysis of the four target oligomers. Hence the standard operating procedure developed by the EURL-FCM was applied by 22 participating laboratories, using HPLC, with acetonitrile-H_2_O gradient programs. Only three laboratories used LC-qTOF-MS or LC–MS/MS techniques, where they applied different mobile phases, and only two laboratories had satisfactory results.

As reported in the questionnaire, the application of MS detection presented slightly lower LODs than the HPLC-UV methods, though different mobile phases and chemical modifiers have been applied. Both laboratories with satisfactory results applied a chemical modifier in their mobile phase (i.e. HCOOH and CH_3_COONH_4_) while the remaining laboratory did not use any modifier and had z- and z'-scores higher than 6.20.

Different types of analytical columns were employed (e.g. C8, phenyl and Envirosep PP columns), with variations of particle size (2.6 up to 5 μm) and length. The predominant stationary phase was C18, followed by Pentafluorophenyl (PFP). The injections volumes, regardless of the different analytical technique used, varied from 8 to 100 μL. Largely discrepant LODs were reported (ranging from 0.001 to 0.050 mg kg^-1^) even by laboratories using the same technique.

## 4 Conclusions

In recent years oligomers have come into the spotlight due to their potential migration from FCMs to food and unknown human health effects. No official analytical methods are yet available and the existence of individual oligomer standards with known purity is very limited. The proficiency test FCM-18-01 organised by the EURL-FCM was a first step in promoting the establishment of comparable measurement data among several laboratories for PES oligomers migrating from FCMs. In fact, to the best of the author's knowledge, it is the first time that a PT has ever been organised for this type of substances at EU level. The overall performance of the participating EU NRLs and OCLs on the determination of PET and PBT cyclic oligomers in official food simulant D1 was satisfactory, despite the lack of experience in this type of analyses for most of the participants. Many of the participants reported underestimated measurement uncertainties. It is highly recommended that laboratories would validate their methods for the determination of oligomers in food simulants.

## Disclaimer

Certain commercial equipment, instruments, and materials are identified in this paper/report to specify adequately the experimental procedure. This does not in no case imply recommendation or endorsement by the European Commission, nor does it imply that the material or equipment is necessarily the best available for the purpose
